# Developmental exposure to DDT or DDE alters sympathetic innervation of brown adipose in adult female mice

**DOI:** 10.1186/s12940-021-00721-2

**Published:** 2021-04-01

**Authors:** Annalise N. vonderEmbse, Sarah E. Elmore, Kyle B. Jackson, Beth A. Habecker, Katherine E. Manz, Kurt D. Pennell, Pamela J. Lein, Michele A. La Merrill

**Affiliations:** 1 Department of Environmental Toxicology, University of California-Davis College of Agricultural and Environmental Sciences, One Shields Avenue, Davis, CA 95616 USA; 2 Department of Molecular Biosciences, University of California-Davis, School of Veterinary Medicine, 1089 Veterinary Medicine Drive, Davis, CA 95616 USA; 3 Present address: Office of Environmental Health Hazard Assessment, California EPA, Oakland, CA USA; 4 Integrative Genetics and Genomics Graduate Group, University of California-Davis, Davis, CA USA; 5 Department of Chemical Physiology and Biochemistry, Oregon Health & Science University, 3181 SW Sam Jackson Park Road, Portland, OR 97239 USA; 6 School of Engineering, Brown University, 184 Hope Street, Providence, RI 02912 USA

**Keywords:** Brown adipose tissue, P,p’-DDE, DDT, Obesogen, Sympathetic innervation, Synaptic connectivity, Thermogenesis, Perinatal exposure

## Abstract

**Background:**

Exposure to the bioaccumulative pesticide dichlorodiphenyltrichloroethane (DDT) and its metabolite dichlorodiphenyldichloroethylene (DDE) has been associated with increased risk of insulin resistance and obesity in humans and experimental animals. These effects appear to be mediated by reduced brown adipose tissue (BAT) thermogenesis, which is regulated by the sympathetic nervous system. Although the neurotoxicity of DDT is well-established, whether DDT alters sympathetic innervation of BAT is unknown. We hypothesized that perinatal exposure to DDT or DDE promotes thermogenic dysfunction by interfering with sympathetic regulation of BAT thermogenesis.

**Methods:**

Pregnant C57BL/6 J mice were administered environmentally relevant concentrations of DDTs (p,p’-DDT and o,p’-DDT) or DDE (p,p’-DDE), 1.7 mg/kg and 1.31 mg/kg, respectively, from gestational day 11.5 to postnatal day 5 by oral gavage, and longitudinal body temperature was recorded in male and female offspring. At 4 months of age, metabolic parameters were measured in female offspring via indirect calorimetry with or without the β3 adrenergic receptor agonist, CL 316,243. Immunohistochemical and neurochemical analyses of sympathetic neurons innervating BAT were evaluated.

**Results:**

We observed persistent thermogenic impairment in adult female, but not male, mice perinatally exposed to DDTs or p,p’-DDE. Perinatal DDTs exposure significantly impaired metabolism in adult female mice, an effect rescued by treatment with CL 316,243 immediately prior to calorimetry experiments. Neither DDTs nor p,p’-DDE significantly altered BAT morphology or the concentrations of norepinephrine and its metabolite DHPG in the BAT of DDTs-exposed mice. However, quantitative immunohistochemistry revealed a 20% decrease in sympathetic axons innervating BAT in adult female mice perinatally exposed to DDTs, but not p,p’-DDE, and 48 and 43% fewer synapses in stellate ganglia of mice exposed to either DDTs or p,p’-DDE, respectively, compared to control.

**Conclusions:**

These data demonstrate that perinatal exposure to DDTs or p,p’-DDE impairs thermogenesis by interfering with patterns of connectivity in sympathetic circuits that regulate BAT.

**Graphical abstract:**

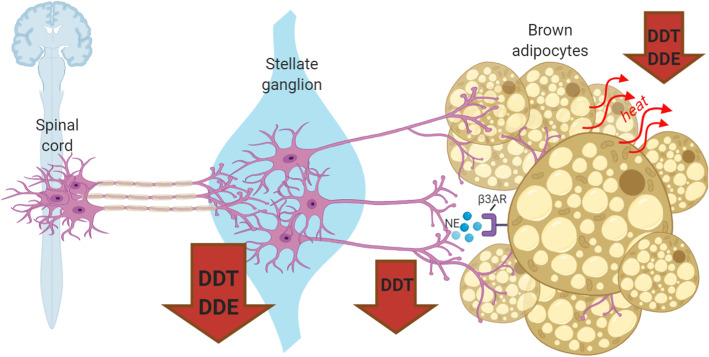

**Supplementary Information:**

The online version contains supplementary material available at 10.1186/s12940-021-00721-2.

## Background

Exposure to the pesticide dichlorodiphenyltrichloroethane (DDT), and its primary metabolite dichlorodiphenyldichloroethylene (DDE), remains a significant public health concern because it is persistent and bioaccumulative and has a long history of widespread global use. Further, exposure is ongoing in many parts of the world because of the recommended use of DDT for malaria control by the World Health Organization, due in part to widespread anti-malarial drug resistance [1]. For example, the median levels of DDT and DDE detected in the blood of immigrants to the USA from India, where DDT continues to be manufactured, exceeded the 95th percentile of DDT and DDE levels in the general U.S. population, and was significantly associated with their increased body mass index and increased odds of obesity in a 2019 study [2].

Exposures to environmental chemicals known as obesogens may play a significant role in the multifactorial etiopathology of obesity, which has become a worldwide epidemic [3–5]. Recent systematic reviews and meta-analyses have concluded that developmental exposure to DDT and DDE is consistently associated with higher body mass index and other measures of adiposity in humans, rats, and mice [2, 6, 7]. For example, perinatal administration of a technical mixture of DDTs (p,p’-DDT and o,p’-DDT) at levels that resulted in human-relevant serum levels of DDTs and p,p’-DDE was associated with reduced energy expenditure and body temperature and increased body fat in adult female mice [8]. Decreased energy expenditure and body temperature are strongly associated with increased susceptibility for obesity [9–13].

Subsequent mechanistic studies of the metabolic effects resulting from perinatal exposure to the technical mixture of DDTs revealed decreased thermogenic signaling in brown adipose tissue (BAT) of 9 month old female mice. The activity of BAT in adult humans is inversely correlated with obesity, diabetes, and insulin resistance [14], and BAT transplantation has been shown to reverse obesity in rodents [15]. Adaptive thermogenesis by BAT is regulated by the sympathetic nervous system (SNS) via norepinephrine (NE) signaling, which stimulates brown adipocytes to generate heat by uncoupling mitochondrial oxidative phosphorylation with an uncoupling protein 1 (UCP1)- mediated proton leak [16]. While the role of adipose dysfunction has been well characterized in studies of DDT and DDE influence on obesity and metabolic disease [17–19], the potential role of DDTs and p,p’-DDE neurotoxicity in initiating or exacerbating these disorders is less clear. The insecticidal activity of DDT is attributed to its interaction with voltage-gated sodium channels to stabilize the channels in their open configuration, resulting in repetitive neuronal firing and hyperexcitability [20]. In addition to this canonical mechanism of neurotoxicity, DDT has been reported to alter in vivo levels of neurotransmitters, including norepinephrine [21], and to inhibit neurite outgrowth in vitro [22, 23]. The potential relevance of these latter observations to the mechanisms by which DDT alters thermogenesis is suggested by rodent studies in which sympathetic denervation of BAT was observed to significantly impair thermogenesis and energy balance coincident with increased body mass [24–29]. But whether developmental administration of DDTs or p,p’-DDE alters the peripheral sympathetic nervous system has not been investigated.

To address this knowledge gap, we leveraged a previously characterized mouse model of developmental exposure to DDTs that caused thermogenic impairment [8], and extended it to include developmental p,p’-DDE exposure. Mice were exposed developmentally to a commercially relevant mixture of p,p’-DDT and o,p’-DDT, or to the in vivo persistent metabolite p,p’-DDE. In addition to measuring various thermogenesis parameters, we quantified synaptic connectivity between pre- and postganglionic sympathetic neurons of the stellate ganglia and between postganglionic sympathetic neurons and BAT. Our data support the hypothesis that DDTs and p,p’-DDE cause persistent defects in adaptive thermogenesis by altering synaptic connectivity of sympathetic neurons that innervate BAT.

## Methods

### Chemicals

The aim of the present study was to determine whether developmental DDT exposures that impair thermogenesis later in life alter sympathetic innervation of BAT. To mimic the typical commercial formulation of DDT prior to its ban in the United States, the DDTs used to dose animals was a combination of 77.2% p,p’-DDT (98.5% purity neat, AccuStandard, New Haven, CT, USA) and 22.8% o,p’-DDT (100% purity neat, AccuStandard). For the DDE exposure, only p,p’-DDE (100% purity neat, AccuStandard) was used. Both p,p’-DDE and the DDTs mixture were separately dissolved in the vehicle (Veh), organic extra virgin olive oil from Italian grown olives (Nugget Markets, Woodland, CA, USA), at a concentration of 1.7 mg/kg (DDTs) and 1.31 mg/kg (p,p’-DDE), which was administered daily by oral gavage (10 μL/kg body weight per day). Dosing solutions were prepared at the beginning of the experiment, stored at room temperature (RT, 21-23 °C) in a gas-tight container, and used within two months. For indirect calorimetry experiments, the β3 adrenergic receptor (AR) agonist CL316,243 (CAS No: 151126–84-0, Cat #1499, 98.1% purity neat; TOCRIS) was dissolved daily in sterile phosphate-buffered saline (PBS, pH 7.4; cat#59321C, Sigma, St. Louis, MO, USA) at a concentration of 0.1 mg/mL and warmed to 37 °C prior to intraperitoneal (i.p.) injection at a volume of 10 μL/g body weight.

Certified reference standards for HPLC quantification were purchased from Accustandard, including o,p’ DDT, p,p’-DDT, o,p’-DDE, p,p’-DDE, ^13^C_12_ labeled p,p’-DDE, D_8_ labeled p,p’-DDT, phenanthrene D-10, and chrysene D-12. Acetone (99.8%, HPLC grade), n-hexane (≥99%), and dichloromethane (99.8%, HPLC grade) were purchased from Fisher Scientific.

### Animal handling and exposures

**Fig. 1:**
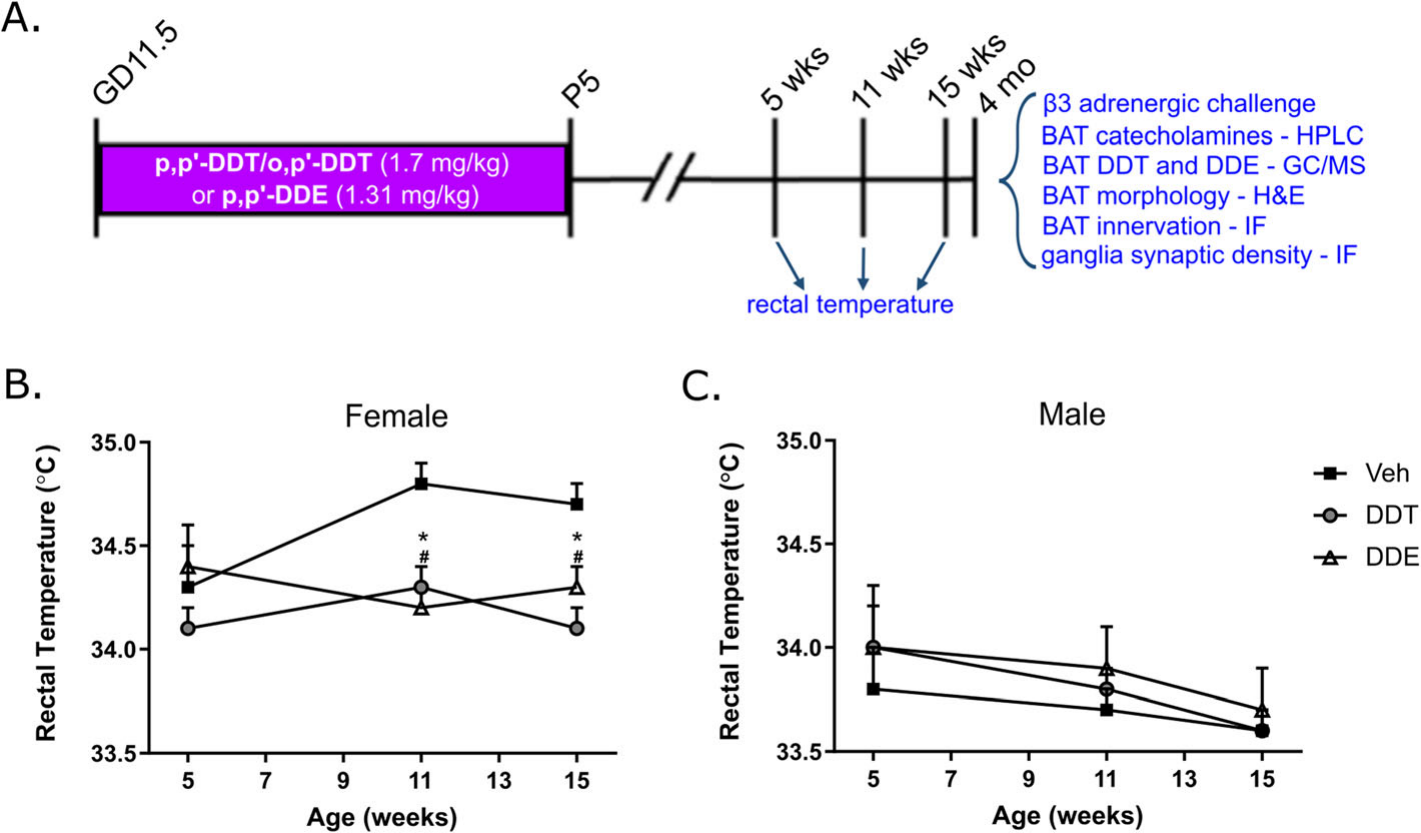
Reduced core body temperature in female mice exposed during early development to DDTs or p,p’-DDE. **a** Schematic diagram of experimental design, including timing of exposure from GD11.5 to P5, and age-specific measurement and analyses in blue. **b**, **c** Core body temperature measured at 5, 11, and 15 weeks in female (**b**) and male (**c**) offspring to assess impairment of thermogenic phenotype in maturing and adult mice. Data presented as mean ± SE, *n* = 14 (VEH), 15 (DDTs) or 7 (p,p’-DDE) non-littermates/group at each time point. Statistical significance (* = DDTs, # = p,p’-DDE) at *p* < 0.05 vs age-matched Veh control. Veh, vehicle (organic olive oil); DDT, dichlorodiphenyltrichloroethane; DDE, dichlorodiphenyldichloroethylene; GD11.5, gestational day 11.5; P5, postnatal day 5; wks, weeks; mo, months; IF, immunofluorescence

Animals were maintained in facilities fully accredited by AAALAC International, and all studies were performed with regard for alleviation of pain and suffering under protocols approved by the UC Davis Institutional Animal Care and Use Committee (IACUC protocol #18938). All animal experiments were conducted in accordance with the ARRIVE guidelines and the National Institutes of Health guide for the care and use of laboratory animals (NIH publication No. 8023, revised 1978). Male and female 8-week-old C57BL/6 J mice were obtained from Jackson Laboratories (Sacramento, CA, USA). Except during mating, adult male and female mice were separately housed with 2–5 animals per cage in standard plastic cages under controlled environmental conditions (12 h light/dark cycle, 40–50% humidity). Litters were kept with the dam until weaned at postnatal day (PND) 21. Room temperature was maintained between 21 and 23 °C, which is considered a minor cold stress with the potential for stimulating low-grade sympathetic activation [30, 31], to mimic thermoregulatory conditions for equivalent metabolic activity in humans [32]. All animals had access to food (5053 Purina diet) and water *ab libitum*. Upon receipt from the vendor (Jackson Laboratories), 8-week-old male and nulliparous female C57BL/6 J mice were acclimated for one week prior to timed mating. Pregnancy was determined by the presence of a postcoital vaginal plug. Primigravid females were randomly assigned to experimental groups using a random number generator, using a priori decisions to maximize litter number per group based on sex per litter; thus, final counts for sex-based longitudinal rectal temperature were *n* = 14 (Veh), 15 (DDTs) or 7 (p,p’-DDE) non-littermates/group for a total of 36 litters, and all remaining analyses in females at *n* = 7 non-littermates/group for a total of 21 litters, unless otherwise stated in the figure or table legends. Once assigned to an experimental group, dams were orally gavaged (10 μL/g body weight) with DDTs (p,p’-DDT and o,p’DDT), DDE (p,p’-DDE), or Veh from gestational day (GD) 11.5 until postnatal day (PND) 5. This period encompasses crucial ontological events for autonomic ganglionic formation, establishment of peripheral sympathetic synaptic connections, sympathetic target tissue innervation, and BAT activation by the sympathetic nervous system [16, 33, 34]. At PND 5, litters were culled to 6 random pups to normalize lactational pesticide transfer, and pups were weaned at PND 21. At 4 months of age, female offspring were euthanized by exsanguination under isoflurane anesthesia. The experimental design is represented schematically in Fig. 1a.

### Temperature measurements and indirect calorimetry

Longitudinal core body temperature was measured in female offspring in four-week intervals beginning at 5 weeks after birth using a thermocoupled probe (RET-4, Physitemp, Clifton, NJ, USA) inserted into the rectum to a depth of 5 mm.

Thermoneutral zone (TNZ) was assessed using indirect calorimetry with implanted temperature recorders. DST nano-T temperature recorders (Star-Oddi, Gardabaer, Iceland) were implanted intraperitoneally under anesthesia (2–4% isoflurane). For analgesia, animals received meloxicam (2–10 mg/kg, subcutaneous, SC) pre-operatively and, as needed, buprenorphine (0.05–0.1 mg/kg, SC) post-operatively. Mice were moved to the calorimetry room at least ten days after surgery. Mice were acclimated to the calorimetry room for 24 h while housed in acclimation chambers. The morning of TNZ analysis, food was removed from the acclimation chamber to ensure that calorimetry readings were not influenced by foraging activity. To evaluate the TNZ, a mouse in an acclimation chamber was transferred to a calorimetry chamber within a temperature-controlled cabinet set at 12 °C. Cage temperature was measured using ambient temperature sensors (DS1922L-F5#, iButtonLink, LLC) attached to the inside of the metabolic cage lid. Previous tests measuring temperature within the calorimeter chambers were used to select the chamber location for the studies that best matched the target temperatures. Energy expenditure was determined by indirect respiration calorimetry [35] recorded every 5 s in the Comprehensive Lab Animal Monitoring Systems (CLAMS, Columbus Instruments) unit at 12 °C for 60 min, at 18 °C, 24 °C, 28 °C, and 30 °C for 45 min each, and at 34 °C and 36 °C for 30 min each. The body temperature recorders were programmed to record every 5 min from the beginning of the 12 °C interval until the beginning of the 30 °C calorimetry measurements and for every minute for the remainder of the calorimetry measurements (30 °C through 36 °C).

Oxygen consumption and heat production in response to CL316,243 was determined by indirect calorimetry [35]. Four-month old female mice were individually housed in CLAMS metabolic chambers and acclimated for at least three days prior to data acquisition. Due to an insufficient number of CLAMS cages for simultaneous, age-dependent analysis of the three exposure groups, indirect calorimetry was performed only on the mice exposed to DDTs or Veh. On the day of pharmaceutical intervention, mice had food and water removed for 4 h prior to intraperitoneal injection (IP) injection with either CL316,243 (0.1 mg/kg) or Veh (sterile phosphate buffered saline [PBS], pH 7.4; #59321C, Sigma-Aldrich, St. Louis, Missouri, USA) by researchers blinded to experimental groups. Subsequent indirect calorimetry metrics were recorded for the next 60 min, with sampling frequency at one min intervals. Measurements were averaged over a 20 min window after chamber gas equilibration, from post-injection minute 11 to 31 to encompass a period of peak BAT activation by CL316,243 [36]. Room temperature was maintained between 21 and 23 °C, rather than at TNZ due to the lack of a DDTs-related shift of TNZ (Supplemental Table 1) and kept on a 12 h light/dark cycle.

### Histology and immunofluorescence

BAT was collected from the intrascapular region immediately following euthanasia. BAT was fixed in 4% (w/v) paraformaldehyde (PFA; Sigma) diluted in 0.2 M phosphate buffer (0.2 M Na_2_HPO_4_, 0.2 M NaH_2_PO_4_, pH 7.2) for 24 h, then transferred to 30% (w/v) sucrose (Sigma) diluted in phosphate buffered saline (PBS; 10 mM Na_2_HPO_4_, 1.76 mM KH_2_PO_4_, 2.7 mM KCl, 137 mM NaCl; pH 7.4; Sigma) for long-term storage at 4 °C. Fixed BAT samples were transferred into 70% v/v ethanol in water, then embedded in paraffin. Three 5 μm thick sections were obtained at 100 μm intervals per tissue sample and stained with hematoxylin and eosin (H&E). H&E-stained sections were analyzed by the UC Davis Comparative Pathology Laboratory (UC Davis School of Veterinary Medicine). A pathologist blinded to study groups examined BAT sections for inflammation, degeneration and necrosis. Vacuolation of BAT adipocytes was subjectively classified as the ratio of cells with small to large intracytoplasmic vacuoles. This quantification avoided obvious tissue edge and blood vessels, and assumed unstained round cellular droplets to be lipids contained within intracytoplasmic vacuoles.

For immunohistochemical analyses, PFA-fixed BAT was embedded in optimal cutting temperature (OCT) compound (#4583, Tissue-Tek, Sakura Finetek, Torrance, CA, USA) and flash-frozen. Three sections per sample were serially sliced 10 μm thick mounted on Superfrost Plus slides (Cat#12–550-15, Fisher Scientific) using a cryostat set to -30 °C (Microm HM550 cryostat, Thermo Scientific), and slides were stored at -80 °C until further processed. Once slides were brought to room temperature (RT), heat-mediated antigen retrieval was performed on slides submerged in citrate buffer (10 mM sodium citrate in 0.05% (v/v) Tween-20 [Sigma-Aldrich] diluted with MilliQ H_2_O, pH 6.0) using a vegetable steamer for 30 min to attain a minimum temperature of 60 °C. Following antigen retrieval, samples were blocked for 1 h at RT in blocking buffer, which was 10% (v/v) normal goat serum (Vector Laboratories, Burlingame, CA, USA), 2% (w/v) bovine serum albumin (Sigma), 0.05% (v/v) Tween-20 in PBS (pH 7.4, Sigma). Sections were then immunoreacted for 48 h at 4 °C with the following primary antibody cocktail diluted in blocking buffer: mouse anti-neuropeptide Y (NPY) (1:250, monoclonal IgG1, ab112473, RRID:AB_10861167; Abcam, Cambridge, MA, USA) and rabbit anti-tyrosine hydroxylase (TH) (1:1000, polyclonal, Cat #AB152, RRID: AB_390204; EMD Millipore, Temecula, CA, USA). After incubation with primary antibodies, sections were washed with PBS containing 0.05% (v/v) Tween-20 and then incubated for 1 h at RT with AlexaFluor-488 goat anti-mouse IgG F(ab’)2 (lot#1812170, Invitrogen, Waltham, MA, USA) and AlexaFluor-568 goat anti-rabbit IgG (lot#1871167, Invitrogen) each diluted to 1:1000 in PBS (pH 7.4) containing 0.05% (v/v) Tween-20. Sections were then washed and coverslipped with ProLong Gold Antifade Mountant with 4′,6-diamidino-2-phenylindole, (DAPI, P36931, ThermoScientific) and left to cure overnight before imaging.

To immunostain stellate ganglia, unfixed stellate ganglia collected immediately upon euthanasia were immediately embedded in OCT (Tissue-Tek) and flash frozen. Samples were serially cryosectioned at 10 μm, permeabilized for 5 min in 0.2% (v/v) Triton X-100, and then blocked for 2 h at RT with blocking buffer. Slides were then immunoreacted overnight at 4 °C with primary antibody cocktail diluted in blocking buffer: rabbit anti-chapsyn-100 (PSD93) (1:500, polyclonal, AB5168-200UL, RRID:AB_91716; EMD Millipore) and mouse anti-bassoon (1:500, ADI-VAM-PS003-F, RRID:AB_11181058; ENZO Life Sciences, Plymouth Meeting, PA, USA). After incubation with primary antibodies, sections were washed with PBS containing 0.02% (v/v) Tween-20 and then incubated for 1 h at RT with AlexaFluor-488 goat anti-mouse IgG F(ab’)2 (lot#1812170, Invitrogen) and AlexaFluor-568 goat anti-rabbit IgG (lot#1871167, Invitrogen) each diluted to 1:1000 in PBS with 0.02% (v/v) Tween-20. Sections were then washed and coverslipped with ProLong Gold Antifade Mountant with DAPI (P36931, Thermo Scientific) and left to cure overnight before imaging. Staining batches were evenly stratified across all treatment groups to minimize batch effects, and appropriate controls were included in each stain batch for downstream inter-batch comparisons and thresholding determinations to optimize signal:noise during quantification.

### Catecholamine concentrations

BAT (*n* = 7/group) was collected immediately after euthanasia and flash frozen. Tissue was homogenized in perchloric acid (300 μl, 0.1 M) containing dihydroxybenzylamine (1.0 μM) internal standard. After homogenization, all samples were centrifuged (13,000 g for 5 min). Catecholamines were purified from an aliquot (100 μL) of the supernatant by alumina adsorption. Norepinephrine (NE) and its metabolite dihydroxyphenylglycol (DHPG) were measured by HPLC with electrochemical detection as described previously [37–39]. Detection limits were ~ 0.05 pmol with recoveries from the alumina extraction > 60%.

### DDT and DDE concentration quantification

Tissue concentrations of o,p’-DDT, p,p’-DDT, o,p’-DDE, and p,p’-DDE were measured in BAT from 4-month-old mice using gas chromatography (GC)-MS following methods described previously [2]. Briefly, 30 mg flash frozen BAT samples (*n* = 3/group) were extracted using a QuEChERS-based extraction with 1:1:1 hexane:acetone:dichloromethane. Briefly, the samples were spiked with 10 μL of a solution containing ^13^C_12_ labeled p,p’-DDE and D_8_ labeled p,p’-DDT as internal standards to assess recovery. The samples and 5 mL of the solvent mixture were added to 7-dram amber vials and vortexed mixed. The supernatant was added to a 15 mL QuEChERS tube containing 150 mg dispersive C18 powder and 900 mg anhydrous MgSO_4_ (United Chemical Technologies, Bristol, PA). The Quechers tube was shaken for 15 min on a rotary shaker (Fisherbrand) and centrifuged (5 min). The supernatant was transfer to a glass centrifuge tube. These steps were repeated two more times with 3 mL of 1:1:1 hexane:acetone:dichloromethane (11 mL total). The final extract was evaporated down to 150 μL under nitrogen (Organomation 30 position Multivap Nitrogen Evaporator). After extraction, samples were spiked with phenanthrene-D10 and chrysene D-12 as internal standards to ensure injection consistency during GC-MS analysis.

Sample extracts were then analyzed on a high-resolution GC Q-Exactive Orbitrap MS (Thermo Scientific) equipped with a Thermo Trace 1300 gas chromatograph and TriPlus RSH Autosampler. The extracts (3 μL) were injected into a 290 °C split/splitless inlet operated in split-less mode. The analytes were separated on a Restek Rxi-35Sil MS column (30 m × 0.25 mm inner diameter × 0.25 μm film thickness) with Helium (99.999% purity) as the carrier gas (1 mL/min). The oven temperature ramp began at 130 °C for 0.5 min, increased 30 °C/min to 235 °C and held for 4 min, 10 °C/min to 275 °C, and 50 °C/min to 320 °C and held for 10 min, with a total run time of 23 min. The transfer line was maintained at 300 °C and the EI source temperature at 250 °C. The MS was operated in full scan mode, with a scan range of 150 to 350 m/z. The most abundant peak in the mass spectrum was used to quantify each analyte and identify was confirmed using the ratio of two confirming ions and retention times (Table 1). Quantification was performed using a ten-point calibration curve prepared by serial dilution of calibration standards in hexane (.007 to 30 μg L^− 1^). The limited of detection (LOD) for each target analyte was determined from seven injections of calibration standards and calculated as previously described [40, 41] using the following equation:

$$ LOD=s\ast t\ \left( df,1-\alpha =0.99\right) $$

where s is the standard deviation of the 7 injections, t is the student’s t-value, df is the degrees freedom, and α is the level of significance (for *n* = 7 and α = 0.01, t = 3.14). Limits of detection (LOD) per metabolite in a 30 mg BAT sample were as follows: 0.026 pg/mg BAT (p,p’-DDT), 0.269 pg/mg BAT (p,p’-DDE), 0.153 pg/mg BAT (o,p’-DDT), and 0.104 pg/mg BAT (o,p’-DDE).

**Table 1:** Retention time and ions monitored to quantify and confirm DDE metabolites

Analyte	RT (min)	Quantifying Ion (m/z)	Confirming Ion 1 (m/z)	Confirming Ion 2 (m/z)
o,p’-DDE	8.36	245.9999	247.9968	317.9345
o,p’-DDT	10.64	235.0076	165.0699	237.0047
p,p’-DDE	9.10	245.9999	247.9968	317.9345
p,p’-DDT	11.35	235.0076	165.0699	237.0047
4,4′-DDE (^13^C_12_)	9.17	260.0370	188.1021	258.0400
4,4′-DDT (D_8_)	11.27	243.0576	173.1200	245.0549

*RT* retention time

### Data processing and analyses

The lower critical temperature (LCT) of the TNZ, previously reported to be between 26 and 32 °C for varying strains of laboratory mice [32, 42], was calculated using a segmental linear model of energy expenditure versus calorimeter chamber temperature with the slope 2 = 0. Although the upper critical temperature (UCT) of the TNZ is frequently defined as the ambient temperature where heat stress induces an increase in energy expenditure [43], we did not consistently observe increases in energy expenditure even at ambient temperatures where body temperature was increased (See Supplementary Table 1, Additional file 1). Therefore, we followed the approach suggested by Abreu-Vieira et al. [44] and used an increase in body temperature to identify the UCT. The UCT was calculated using a segmental linear model of core body temperature versus calorimetry chamber temperature with the slope 1 = 0. Energy expenditure in mice was measured at temperatures ranging from 12 to 36 °C, after which Scholander plots were constructed [45] to determine the effect of ambient temperature on energy expenditure and identify the TNZ (Table S1). Only body temperature data collected at calorimetry chamber temperatures above 28 °C were used for the calculations to avoid fluctuations in body temperature that were more common at colder temperatures.

Images of immunofluorescence were captured using an Olympus IX81 wide-field microscope with either a 20x SAPO BF or 60x SAPO BF objective lens for BAT and stellate, respectively, with consistent exposure times across all samples. Regions of interest (ROIs) were randomly selected per tissue section (*n* = 1–3 ROI technical replicates within each section, depending on the availability of non-overlapping viewing fields and tissue size) for each of the three tissue sections per slide, for a total of *n* = 3–9 ROIs per sample evaluated. Automated, nonbiased colocalization analyses were performed using MetaXpress Image Analysis Software (MetaXpress Image Acquisition and Analysis Software v6.1, Molecular Devices Corp., USA) based on a priori-designated signal:noise ratios per channel and background fluorescence thresholds for tissue-specific immunopositive staining. Target-specific controls lacking primary or secondary antibodies were included in each staining batch to determine thresholds for immunopositive fluorescence and adjust for batch effects.

All data were presented as the mean ± standard error of the mean (SE). Data were assessed for normality using Kolmogorov-Smirnov or Shapiro-Wilk normality tests, and outliers were removed based on identification via Grubb’s test (GraphPad Prism version 8.4.0, GraphPad Software Inc., San Diego, CA, USA). Histological analyses and catecholamine measurements were then assessed using either nonparametric Kruskal-Wallis tests with Dunn’s correction for data not normally distributed (e.g. H&E histology data) or analysis of variance (ANOVA) with Tukey’s correction for normally distributed data (e.g. immunofluorescence and catecholamine outcomes) using GraphPad PRISM 7. Body temperature was normally distributed and thus least square means differences between categorical effect of perinatal treatment were analyzed at each age using general linear models (PROC GLM, SAS v9.4, Statistical Analysis System (SAS) Institute, Cary, NC, USA). Indirect calorimetry-derived parameters were modeled with the fixed effects of perinatal exposure, acute (agonist or Veh) exposure, and their interaction, while accounting for the repeated measures from individual mice and resulting covariance structure in a mixed linear model that allows the data to exhibit within- individual correlation (PROC MIXED, SAS v9.4). All parameters were statistically analyzed at a significance threshold of *p* < 0.05 for main effects and *p* < 0.1 for interaction effects.

## Results

It was previously reported that perinatal exposure to a mixture of DDTs caused significant defects in thermogenesis in adult female mice that increased in severity with age [8]. To confirm and further extend these earlier findings, we examined the effects on thermogenesis of perinatal exposure to not only DDTs (which results in a mixed exposure to DDT and DDE due to metabolism of DDTs), but also to p,p’-DDE only in adult male and female mice. We found toxicant-related thermogenic impairment that manifested in adulthood among female mice exposed to either DDTs or p,p’-DDE (Fig. 1). Specifically, while no significant toxicant-related differences in body temperature were present in either sex at 5 weeks of age, by 11 weeks exposure to either DDTs or p,p’-DDE significantly reduced female body temperature compared to Veh controls (Fig. 1b, Supplemental Table 2). The female-specific temperature defect persisted out to 15 weeks in both the DDTs and p,p’-DDE exposure groups (Fig. 1b, Supplemental Table 2). In contrast, body temperature of male offspring was unaltered by early-life exposure to DDTs or p,p’-DDE up to 15 weeks of age (Fig. 1c, Supplemental Table 2), confirming the previously reported sex bias in DDTs-induced thermogenic impairment [8].

**Fig. 2:**
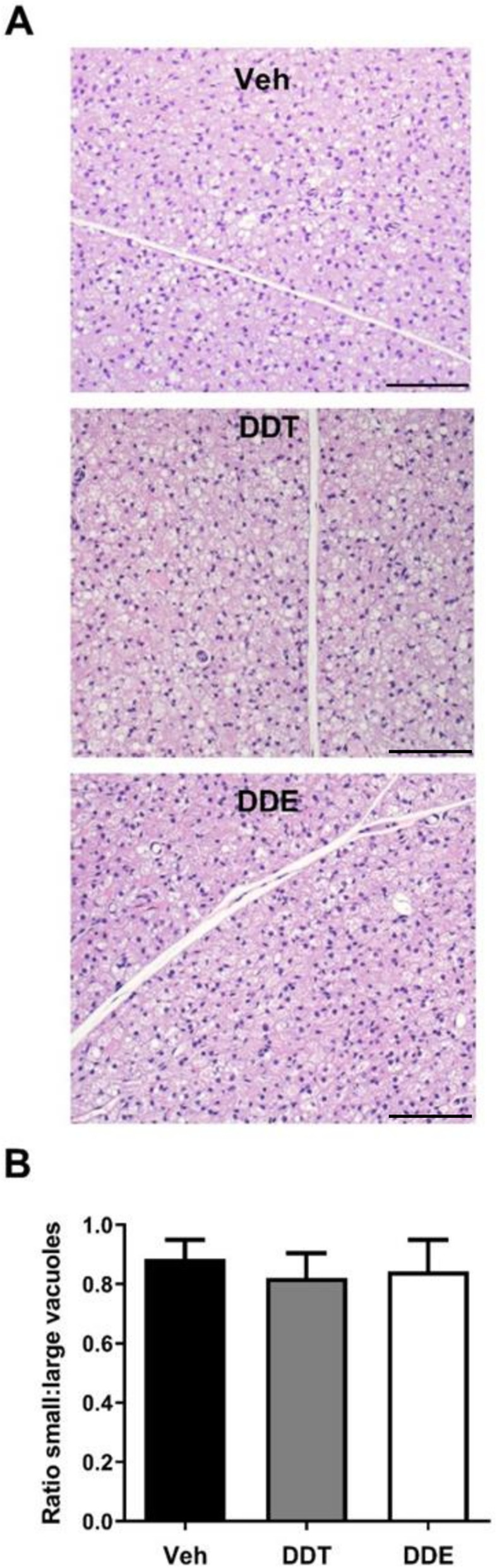
Early-life exposure to DDTs or p,p’-DDE did not alter BAT morphology in adult female mice. **a** Representative images of H&E-stained BAT from 4-month-old female mice. Scale bar = 100 μm. **b** Quantification of H&E-stained BAT morphology as the ratio of cells with small to large vacuoles, *n* = 3 sections per sample, with *n* = 7 animals per group used to calculate mean values. All data presented as mean ± SE. No significant differences between groups were identified using one-way ANOVA with p set at 0.05. Veh, vehicle (organic olive oil); DDT, dichlorodiphenyltrichloroethane; DDE, dichlorodiphenyldichloroethylene

Reduced thermogenesis could be further revealed by increased vacuolization of multilocular brown adipocytes, which are reminiscent of adipocytes with lowered thermogenic capacity [46–48]. To determine whether perinatal DDTs or p,p’-DDE exposure altered vacuolization of brown adipocytes, H&E histochemical staining of adult BAT was performed. The ratio of small to large lipid vacuoles, a quantitative metric of brown adipocyte multilocularity [47, 48], was unaffected by either DDTs or p,p’-DDE (Fig. 2b), indicating no abnormal lipid storage or “whitening” of brown adipocytes that may have contributed to the overall metabolic phenotype at this age. Indeed, no changes to BAT morphology as a result of perinatal DDTs- or p,p’-DDE- exposure were apparent by light microscopy (Fig. 2a).

**Fig. 3:**
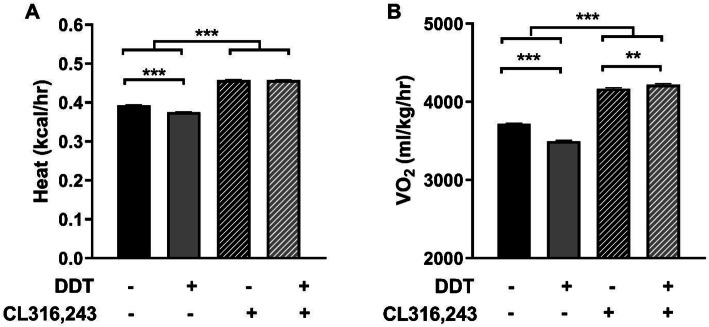
Perinatal exposure to DDTs alters metabolism in 4-month-old female mice. Indirect calorimetry measurements for **a** heat and **b** oxygen consumption following IP injection with β3AR agonist CL 316,243 (0.1 mg/kg) or PBS, recorded and averaged from minute 11 to minute 31 post-injection. Data presented as mean ± SE, n = 7 animals per group. Indirect calorimetry-derived parameters were modeled with the fixed effects of perinatal exposure, acute (agonist or Veh) exposure, and their interaction, while accounting for the repeated measures from individual mice and resulting covariance structure in a mixed linear model that allows the data to exhibit within- individual correlation (PROC MIXED, SAS v9.4). Significance thresholds were p < 0.05 for main effects and *p* < 0.1 for interaction effects, vs Veh. ***p < 0.001; **p < 0.01. DDT, dichlorodiphenyltrichloroethane

Previously, perinatal exposure to DDTs significantly reduced adaptive energy expenditure and thermogenesis in six-month-old female mice [8]. To determine if this effect was BAT-autonomous, we tested whether mimicking sympathetic stimulation of adaptive thermogenesis by administering the β3AR agonist CL316,243 [49] rescued the effects of DDTs on respiration. Perinatal DDTs exposure reduced basal heat production in 4-month-old female mice (Fig. 3a), as previously observed in six-month-old female mice exposed to an identical dose and mixture of DDTs [8]. Basal oxygen consumption, an indirect measure of metabolism [42], was also reduced in four-month-old mice exposed perinatally to DDTs relative to controls (Fig. 3b). Metabolic impairment was observed in the absence of significant differences in physical activity between Veh control and DDTs mice (See Supplemental Fig. 1). Direct stimulation of brown adipocytes by CL 316,243 rescued measures of indirect calorimetry in DDTs-exposed animals with a significant interaction between acute CL 316,243 treatment and perinatal DDTs. Heat production was lower in DDTs-exposed mice compared to controls, but DDTs animals treated with CL 316,243 responded at the same level as Veh control animals (Fig. 3a). Likewise, basal oxygen consumption in DDTs-exposed animals treated with CL 316,243 were similar to Veh controls (Fig. 3b).

**Fig. 4:**
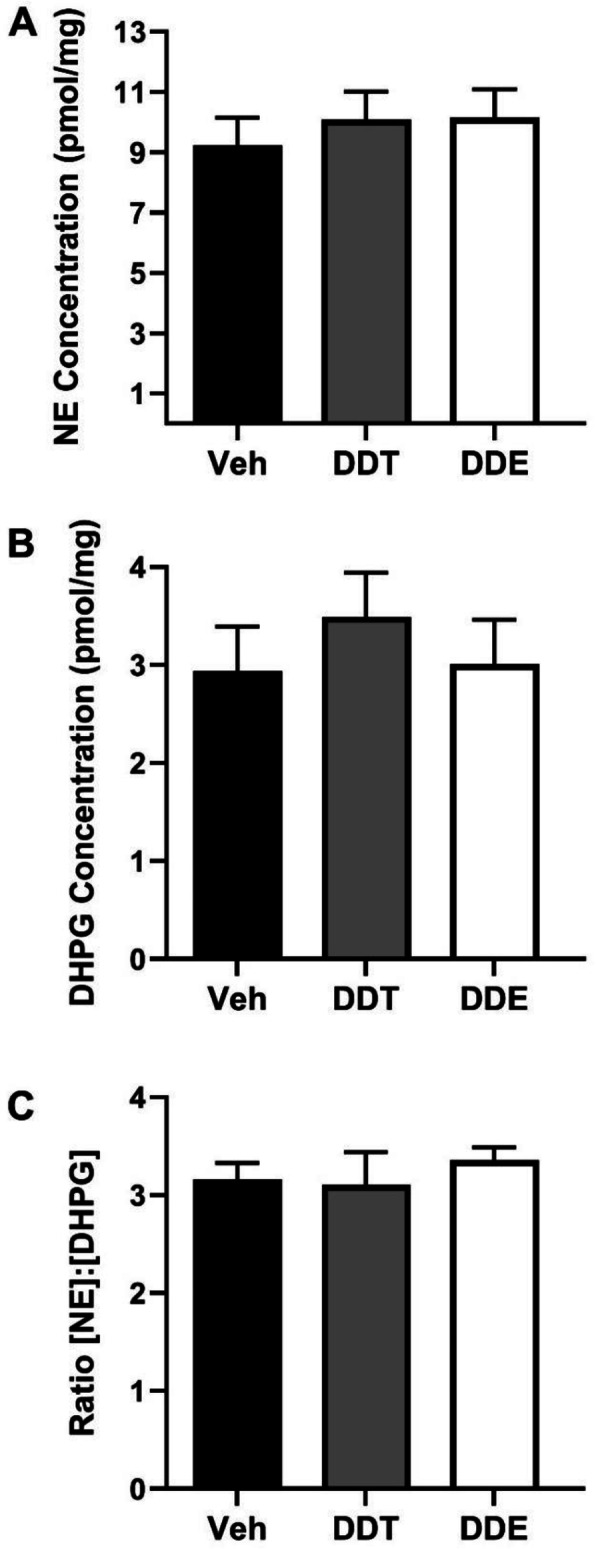
Perinatal DDTs or p,p’-DDE exposure doesn’t significantly alter BAT catecholamine levels in adult female mice. Concentration of **a** norepinephrine (NE), its metabolite **b** DHPG, and **c** the ratio of NE:DHPG in BAT were determined using HPLC from n = 7 animals per treatment, age 4 months. No significant differences between groups were identified using ANOVA with p set at 0.05. BAT, brown adipose tissue; Veh, vehicle (organic olive oil); DDT, dichlorodiphenyltrichloroethane; DDE, dichlorodiphenyldichloroethylene; NE, norepinephrine; DHPG, dihydroxyphenylglycine

The observation that acute stimulation of β3-adrenergic receptors with CL (Fig. 3) restored normotypic metabolic responses in female mice exposed perinatally to DDTs suggested that DDTs target mechanisms that regulate BAT metabolism upstream of the β3-adrenergic receptor, such as catecholamines [50, 51]. Thus, we next quantified total concentrations NE and the primary NE metabolite, DHPG in BAT to examine whether effects of DDTs on respiration (Fig. 3) were mediated by decreased release of norepinephrine from sympathetic nerve terminals innervating BAT. HPLC analysis revealed no detectable differences in total NE (Fig. 4a) or DHPG (Fig. 4b) concentrations in BAT from control versus DDTs- or p,p’-DDE -exposed female mice (Fig. 4a). The ratio of [NE]:[DHPG] was also evaluated as a metric of intraneuronal turnover [52], but similarly did not reveal significant differences between groups (Fig. 4c).

**Fig. 5:**
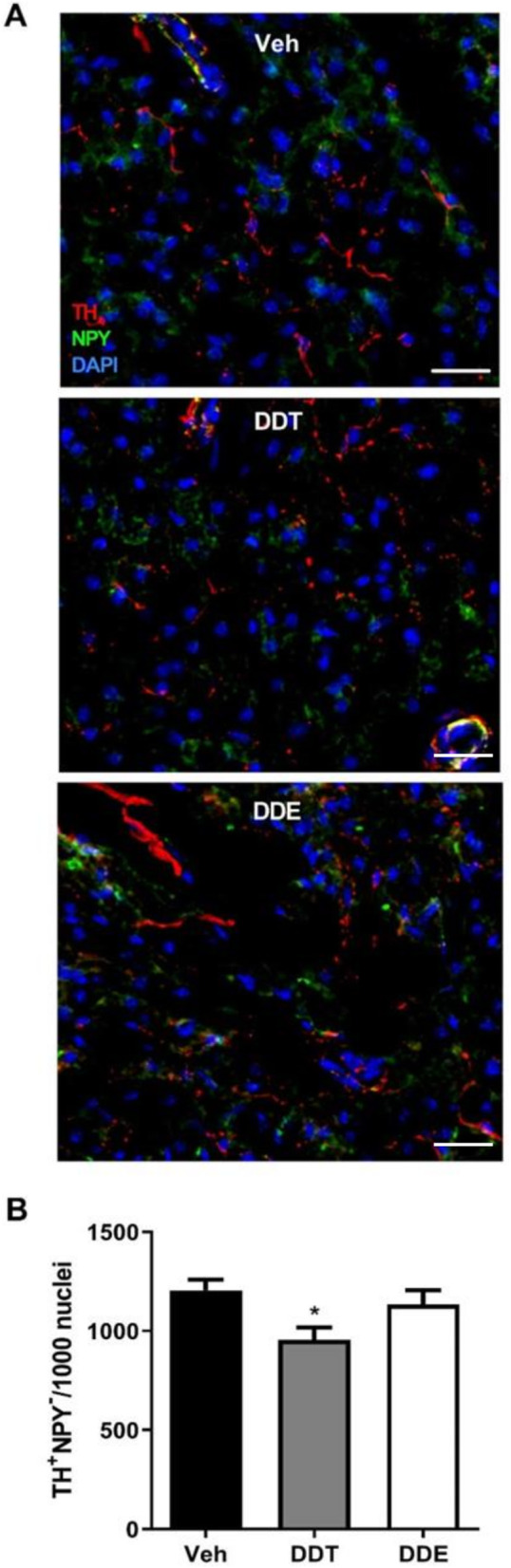
Perinatal exposure to DDTs decreased sympathetic innervation of BAT in adult female mice. **a** Representative images of BAT immunostained for tyrosine hydroxylase (TH, red), neuropeptide Y (NPY, green), and counterstaining with DAPI to identify cell nuclei. Fibers immunopositive for TH but not NPY innervate BAT; fibers immunopositive for both TH and NPY innervate blood vessels. Scale bar = 25 μm. **b** Quantification of adrenergic innervation as determined by % area TH+/NPY- per ROI, with *n* = 4–8 ROIs averaged per sample and *n* = 6–7 animals per treatment used to calculate the mean. Data represented as mean positive immunoreactivity per ROI normalized per 1000 DAPI+ nuclei ±SE. Statistical significance determined by one-way ANOVA with post hoc Tukey’s test. *p < 0.05 vs Veh. BAT, brown adipose tissue; Veh, vehicle (organic olive oil); DDT, dichlorodiphenyltrichloroethane; DDE, dichlorodiphenyldichloroethylene

The absence of an obvious neurochemical aberration in BAT (Fig. 4) but the rescue of DDTs-impaired respiration by pharmacologic agonism of the β3 receptor (Fig. 3) suggested the possibility that perinatal DDTs interfere with morphologic determinants of sympathetic innervation of BAT. To address this, we first investigated the effects of perinatal DDTs and p,p’-DDE on the density of sympathetic axons in BAT from adult female mice. BAT was immunostained for tyrosine hydroxylase (TH) and neuropeptide Y (NPY) to distinguish sympathetic axons that innervated brown adipocytes (TH immunopositive) from those that innervated the perivascular system (immunopositive for both TH and NPY) [53, 54]. The DDTs exposure significantly reduced sympathetic innervation in 4-month-old BAT by approximately 20% relative to control levels (Fig. 5b, Supplemental Table 2). Decreased innervation of BAT appeared to be driven by DDTs specifically as innervation of p,p’-DDE -exposed BAT was not significantly different from that of Veh-exposed mice (Fig. 5).

**Fig. 6:**
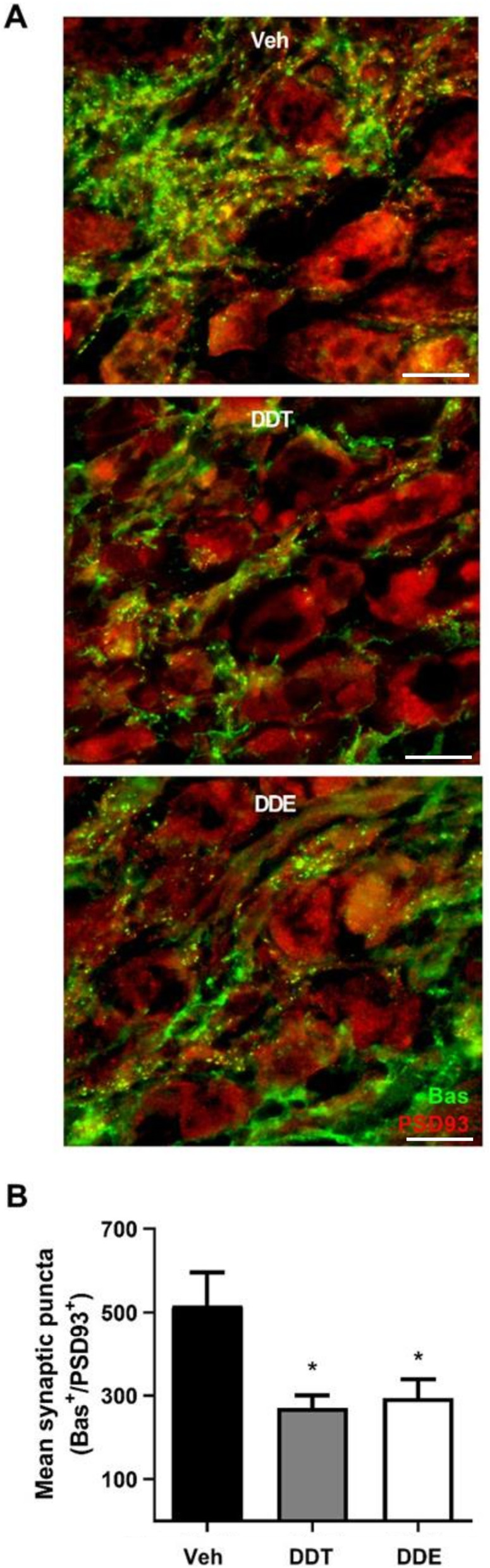
Perinatal DDTs and p,p’-DDE exposure decreased synaptic density in stellate ganglia of 4-month-old female mice. **a** Representative images of stellate ganglia stained for synaptic puncta, identified by co-localization of the presynaptic marker Bassoon (Bas, green) and the postsynaptic marker PSD93 (red). Scale bar = 14 μm. **b** Quantification of mean synaptic puncta per ROI, with n = 6–9 ROIs averaged per sample and 4–5 animals per treatment used to calculate the mean. Data presented as mean ± SE. Statistical significance determined by one-way ANOVA with post hoc Tukey’s test. *p < 0.05 vs Veh. Veh, vehicle (organic olive oil); DDT, dichlorodiphenyltrichloroethane; DDE, dichlorodiphenyldichloroethylene; Bas, Bassoon; PSD93, postsynaptic density 93

Sympathetic tone is determined not only by the density of sympathetic axons innervating target tissue, but also by the density of synaptic contacts between pre- and post-ganglionic neurons [55, 56]. BAT is predominantly innervated by the stellate ganglia [16, 57]; therefore, we quantified synaptic connectivity in stellate from adult female mice via immunohistochemical co-localization of the presynaptic marker, Bassoon (Bas), and the postsynaptic marker, postsynaptic density 93 (PSD93) [58, 59]. Perinatal exposure to either DDTs or p,p’-DDE reduced synaptic density in the stellate ganglia by approximately 48 and 43%, respectively, compared to controls (Fig. 6, Supplemental Table 2).

DDT and DDE concentrations in BAT in the current study were quantified in male offspring at 4 months post-exposure. The primary metabolite detected in the DDTs-administered group was p,p’-DDT, at a low concentration of 1.518 ng/g BAT (Table 2). A roughly 10% reduction in the proportion of o,p’-DDT was detected than was administered, consistent with its more rapid metabolism. BAT from p,p’-DDE-administered mice showed even less accumulation, with only 0.570 ng/g of the most concentrated metabolite, p,p’-DDE, accounting for nearly 74.3% of all measured DDT and DDE isoforms. p,p’-DDE was found only in BAT of mice for which it was administered. Consistent with the degradation of p,p’-DDE into o,p’-DDE [60, 61], some o,p’-DDE was also detected.

**Table 2:** DDT metabolite concentration in brown adipose of 4-month-old female mice perinatally exposed to DDTs or p,p’-DDE

	DDTs dose group	p,p’-DDE dose group
	(n = 3)	(n = 3)
Measured Metabolite	mean ± SE (ng/g BAT)	mean ± SE (ng/g BAT)
p,p’-DDT	1.518 ± 0.373	0.027 ± 0.001
p,p’-DDE	<LOD	0.570 ± 0.066
o,p’-DDT	0.217 ± 0.024	<LOD
o,p’-DDE	<LOD	0.170 ± 0.024

BAT = brown adipose tissue; SE = standard error; LOD = limit of detection.

## Discussion

In this work we demonstrate that oral gavage with p,p’-DDE alone mimics thermogenic effects previously reported in 6-month-old female mice with combined perinatal exposure to DDTs [8], which we now show can be observed as early as 11 weeks after birth. While prevailing hypotheses of the mechanism(s) by which environmental obesogens alter metabolism have primarily focused on physiological and metabolic dysfunction in adipose tissue; our findings identify modulation of synaptic connectivity in sympathetic circuits that regulate BAT as an additional, novel mechanism contributing to the effects of DDTs and p,p’-DDE on thermogenesis.

The observation that administration of DDTs or p,p’-DDE during the perinatal period each persistently reduced thermogenesis in adult female mice is consistent with reports that obesity is correlated with exposures to DDT or DDE [4, 6, 62] and with reduced thermogenesis [63, 64]. While exposure to mixtures of DDTs and p,p’-DDE have been previously shown to reduce the body temperature of adult mice and rats [8, 62], this is the first study to demonstrate that administration of p,p’-DDE alone can cause such an effect.

Our data strongly support the hypothesis that the thermogenic defect observed in female mice perinatally exposed to DDTs or p,p’-DDE is mediated by perturbation of sympathetic regulation of BAT. The most direct evidence of this is the observation that pharmacologic agonism of β3 adrenergic receptors rescued DDTs-related impairments in heat production and oxygen consumption. β3 adrenergic receptors are highly expressed in brown adipose tissue where they function to regulate lipolysis [16], as indicated by reports of decreased oxygen consumption in NE-stimulated β3-KO mice [65]. Collectively, these observations suggest that DDTs impair thermogenesis by targeting mechanisms of thermogenesis upstream of adipose tissue. Unfortunately there was no remaining BAT to evaluate its UCP1 expression, a protein predominantly responsible for the tissue’s thermogenic properties [66], but future studies should examine whether DDTs or p,p’-DDE cause brown adipocyte-autonomous uncoupling of respiration.

BAT thermogenesis is regulated by the sympathetic nervous system [16], and a recent report demonstrates that BAT thermogenesis can be modulated by influences on peripheral sympathetic neurons independent of input from CNS centers [67], Sympathetic tone at the level of the target tissue is strongly influenced by the density of sympathetic innervation of the target tissue and by the extent of dendritic arborization of the postganglionic neurons [68]. As determined using quantitative immunohistochemistry, sympathetic innervation of BAT was significantly reduced in four-month-old female mice perinatally exposed to DDTs. However, a similar effect was not observed in p,p’-DDE-exposed animals. In contrast, perinatal exposure to either DDTs or p,p’-DDE significantly reduced synapse density in stellate ganglia of four-month-old female mice. There is a direct relationship between synapse density in sympathetic ganglia and the size of the dendritic arbor of postganglionic sympathetic neurons [57, 69]. Therefore, the significantly reduced synapse density in stellate ganglia from female mice perinatally exposed to either DDTs or p,p’-DDE is an indication that developmental exposure to these chemicals decreases dendritic arborization in stellate sympathetic neurons. Collectively, our data suggest that DDTs and p,p’-DDE impair thermogenesis via convergent (decreased dendritic arborization of postganglionic neurons) and divergent (decreased innervation of BAT) sympathetic mechanisms. This is consistent with literature demonstrating that axons and dendrites are differentially regulated, particularly during development [68]. Whether DDTs and p,p’-DDE interfere with the synaptic connectivity of peripheral sympathetic neurons via direct neurotoxic effects on neurons or by altering target influences on dendritic and axonal morphogenesis of sympathetic neurons [68, 70] remains to be determined.

DDT and DDE differ in the potency of their interactions with sex steroid receptors with o,p’DDT exhibiting 7-10x greater potency at the estrogen receptor (ER) [71–73], and with p,p’-DDE considered primarily an antagonist of the androgen receptor [74–76]. It is unlikely that androgen receptor antagonism influenced the sympathetic thermogenic program given prenatal testosterone decreases heat production in adult female mice [77]. However, there is extensive evidence that estrogen modulates peripheral sympathetic neuroeffector activity related to the thermogenic program [78–80]. For example, reduced sympathetic innervation of the vasculature by decreasing levels of nerve growth factor (NGF) in both the sympathetic ganglia and target tissue [81]. This suggest the possibility that disparate effects between developmental exposure to DDTs versus p,p’-DDE exposure groups on sympathetic innervation of BAT may be mediated the pro-estrogenic signaling of o,p’DDT to lower relevant tissue levels of NGF in DDTs-exposed groups. While this hypothesis has yet to be investigated, ER activation likely does not contribute to effects on thermogenesis or synaptic density in stellate ganglia since these outcomes were observed in female mice exposed developmentally to either the DDTs or p,p’-DDE.

Adaptive thermogenesis by BAT is triggered by release of NE from postganglionic sympathetic neurons [82], and NE levels in BAT under steady-state conditions have historically been used as a direct index of SNS innervation of BAT [83]. We did not observe any significant effects of DDTs or p,p’-DDE on NE or DHPG levels in BAT from 4-month old female mice. Quantification of NE turnover as indicated by the ratio [NE]:[DHPG] similarly revealed no significant DDTs effect. The reason for the apparent disconnect between DDTs-related effects on morphologic determinants of synaptic connectivity versus NE levels in BAT are unknown. One possibility is that under basal conditions, postganglionic sympathetic neurons in adult female mice perinatally exposed to DDTs synthesize and/or release more NE than Veh control animals to compensate for decreased synaptic density, but they are unable to respond appropriately to thermogenic triggers. Arguing against this possibility is a previous report that perinatal exposure to environmentally relevant levels of DDT decreases NE levels in the systemic circulation of prepubertal and adult rats [21]. A second possibility is the potential contribution of NE from sources other than sympathetic neurons, such as BAT-associated macrophages and paracrine signaling from white adipocytes [84, 85]. While the lack of effect on NE and DHPG levels in BAT suggest that DDTs and p,p’-DDE do not impair thermogenesis by altering NE synthesis, release, or uptake in sympathetic terminals that innervate BAT, further studies are needed to confirm this conclusion.

Tonic activity in postganglionic sympathetic neurons is strongly correlated with dendritic arborization [55, 86]. Thus, the significant decrease in synaptic density observed in stellate ganglia of DDTs and p,p’-DDE-exposed mice suggests that these perinatal exposures led to persistent attenuation of tonic activity and subsequently impaired thermogenic signaling. To our knowledge, this is the first evidence to support the hypothesis that administration of DDTs and p,p’-DDE impair thermogenesis by reducing synaptic density in sympathetic circuits that regulate BAT. This proposed mechanistic model is supported by previous reports that sympathetic denervation of BAT significantly impaired thermogenesis and energy balance coincident with increased body mass in rodents [24–29]. Conversely, an increased number of sympathetic ganglionic cells innervating BAT has been reported in mice postnatally exposed to cold (18 °C), which correlated with increased NE concentration in BAT and two-fold higher sympathetic nerve activity to BAT [87].

Despite no observed changes to the thermoneutral zone or NE levels in BAT, basal heat production was attenuated in female mice exposed to DDTs versus Veh while also exposed to mild cold stress (21-23 °C). Impairments in the expected minor sympathetic activation of BAT under mild cold stressed [31] may have further limited the thermogenic capacity of females administered DDTs. Future studies might utilize thermoneutrality as an environmental temperature at which sympathetic input to BAT is minimal [88] to further explore sex-specific thermogenic sympathetic activity and its relation to DDTs- and p,p’DDE-related female susceptibility.

The observation that p,p’-DDE alone reduces body temperature is relevant to long term human exposures. Due to their lipophilicity, DDTs and p,p’-DDE persistently accumulate in adipose tissue, with human elimination half-lives estimated to be 2.1 years and 7.6 years, respectively [89]. DDE concentrations observed in BAT of four-month-old female mice exposed perinatally to p,p’-DDE were over ten-fold lower than p,p’-DDE levels in the adipose tissue (median p,p’-DDE concentrations of 5.64 ng/g and 9.17 ng/g in visceral and subcutaneous adipose tissue, respectively) of adolescents undergoing bariatric surgery in Cincinnati, Ohio, where p,p’-DDE exposure presumably comes from diet and the environment rather than metabolized p,p’-DDT [90]. In addition, DDT and DDE concentrations observed in BAT of four-month-old female mice exposed perinatally to DDTs and p,p’-DDE were 4000 to 27,000 times, respectively, lower than levels in the adipose (5910 ng/g maternal subcutaneous adipose for the sum of DDT and metabolites) of Kenyan mothers immediately after birth [91], which could have resulted from the metabolism of DDT into DDE in addition to direct DDE exposure. The comparatively low levels of accumulated DDTs and p,p’-DDE in BAT of four-month-old mice corroborate the hypothesis that even low levels of exposures are associated with persistent thermogenic impairment of BAT.

## Conclusions

The data presented herein support the hypothesis of developmental origins of health and disease (DOHaD) in the context of metabolic dysfunction and obesity [92]. Specifically, we describe novel data indicating that perinatal exposure to DDTs or p,p’-DDE interferes with the development of the sympathetic circuits that regulate BAT thermogenic activity in adulthood. While various environmental exposures and altered conditions during the perinatal period have been shown to cause sustained changes in the neural regulation of thermogenic systems in animal models [83, 87, 93, 94], ours is the first study to implicate changes in sympathetic neuromodulation as a mechanism contributing to the effects of perinatal exposure to DDTs or p,p’-DDE on thermogenic impairment later in life. Further, in contrast to the traditional epigenetic focus of many DOHaD studies [95–97], these data add to a growing literature identifying the reprogramming of neural circuitry, in this case, altered wiring of the peripheral nervous system, during a critical window of development as an alternative mechanism in the developmental origins of environmentally-induced obesity.

## Supplementary Information

**Additional File 1: Supplemental Table 1.** Analysis of Thermoneutral Zone. **Supplemental Fig. 1.** Quantification of mouse physical activity. **Supplemental Table 2.** Summary of key results by exposure

## Data Availability

The datasets used and/or analyzed during the current study are available from corresponding author on reasonable request.

## Abbreviations

AR: adrenergic receptor
BAT: brown adipose tissue
CLAMS: comprehensive lab animal monitoring system
DAPI: 4′,6-diamidino-2-phenylindole,
DDT: dichlorodiphenyltrichloroethane
DDE: dichlorodiphenyldichloroethylene
DHPG: dihydroxyphenylglycine
DIT: developmental impaired thermogenesis
DOHaD: developmental origins of health and disease
GC: gas chromatography
GD: gestational day
HPLC: high performance liquid chromatography
IACUC: institutional animal care and use committee
IP: intraperitoneal injection
LCT: lower critical temperature
LOD: limits of detection
MS: mass spectrometry
NE: norepinephrine
NPY: neuropeptide Y
OCT: optimal cutting temperature
PBS: phosphate-buffered saline
PFA: paraformaldehyde
PND: postnatal day
PSD93: postsynaptic density 93
ROI: region of interest
RT: room temperature
SC: subcutaneous
SE: standard error of the mean
SNS: sympathetic nervous system
ST: stellate ganglia
UCP1: uncoupling protein 1
UCT: upper critical temperature
T2D: type II diabetes
TH: tyrosine hydroxylase
TNZ: thermoneutral zone
Veh: vehicle
