# Supplementary material for: Developmental exposure to DDT or DDE alters sympathetic innervation of brown adipose in adult female mice
Source: Environ Health. 2021 Apr 1;20:37. doi: 10.1186/s12940-021-00721-2 (PMC8017793; doi:10.1186/s12940-021-00721-2)
Supplement: Supplementary file 1 — Additional File 1: Supplemental Table 1. Analysis of Thermoneutral Zone. Supplemental Fig. 1. Quantification of mouse physical activity. Supplemental Table 2. Summary of key results by exposure [file 12940_2021_721_MOESM1_ESM.docx]

**Additional File 1**

| **Temperature** | **Duration** |
| --- | --- |
| **12C** | 60min |
| **18C** | 45min |
| **24C** | 45min |
| **28C** | 45min |
| **30C** | 45min |
| **32C** | 30min |
| **34C** | 30min |
| **36C** | 30min |

**Supplemental Table 1. Analysis of Thermoneutral Zone**. Parameters (left table) and detailed timing (right) for TNZ assessment. Altered TNZ was defined as an increase in energy expenditure at temperatures where body temperature was also increased. Energy expenditure was evaluated by indirect respiration calorimetry via CLAMS units at 12 °C for 60 min, at 18, 24, 28, and 30 °C for 45 min each, and at 34 and 36 °C for 30 min each to identify the upper critical temperature (UCT) of the TNZ, defined as the temperature where energy expenditure increases. The body temperature recorders were programmed to record every 5 minutes from the 12° C until the beginning of the 30° C calorimetry measurements and for every minute for the remainder of the calorimetry measurements (30° C through 36° C).

| **Outcome** | **Baseline** | **CL** | **TNZ** |
| --- | --- | --- | --- |
| Rm temp | 22°C | 22°C | see table at right |
| Duration | ~72 hr | ~1hr | see table at right |
| Start | Fri AM | ~10:00-14:00 | ~6:30 |
| Stop | Mon AM | ~11:00- 15:00 | ~12:30 |
| food status | free | ~3hr from start | At start of TNZ |
| interval CLAMS | 12min | 5s | 5s |


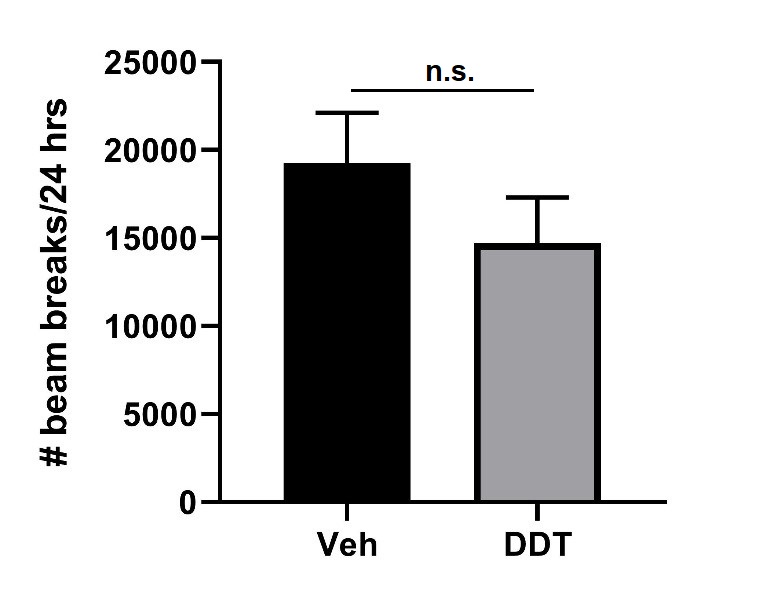


**Supplemental Figure 1. Perinatal DDT does not alter physical activity in 4 month old female mice.** Locomotor activity measured as number of beam breaks over 24 h during indirect calorimetry. Data are expressed as mean SEM; n= mice per treatment group. Statistical analysis performed using unpaired t-test with Welch’s correction. DDT, dichlorodiphenyltrichloroethane.

**Supplemental Table 2. Summary of key results by exposure**. Data presented as mean ±SEM. *p<0.05 denotes statistical significance compared to sex-matched vehicle control (Oil). Abbreviations: Bas, Bassoon; BAT, brown adipose tissue; DDT, dichlorodiphenyltrichloroethane; DDE, dichlorodiphenyldichloroethylene; NPY, Neuropeptide Y; PSD93, postsynaptic density 93; ST, stellate ganglia; TH, tyrosine hydroxylase; Veh, vehicle control; wks, weeks.

|  | **Rectal temp (⁰C)** | | **BAT innervation** | **ST synaptic density** |
| --- | --- | --- | --- | --- |
|  | 11 wks | 15 wks | TH+/NPY- per 1000 nuclei | mean # puncta (Bas+/PSD93+) |
| **Oil** (Veh) | ♀ 34.8 ±0.1 | ♀ 34.7 ±0.1 | ♀ 1206 ±53.59 | ♀ 517 ±79.52 |
|  | ♂ 33.7 ±0.1 | ♂ 33.6 ±0.1 |  |  |
|  |  |  |  |  |
| **DDTs** (o,p' & p,p') | ♀ 34.3 ±0.1* | ♀ 34.1 ±0.1* | ♀ 955.1 ±63.01* | ♀ 270.8 ±29.78* |
|  | ♂ 33.8 ±0.1 | ♂ 33.6 ±0.1 |  |  |
|  |  |  |  |  |
| **p,p'-DDE** | ♀ 34.2 ±0.1* | ♀ 33.6 ±0.1* | ♀ 1133 ±72.17 | ♀ 293.6 ±45* |
|  | ♂ 33.9 ±0.2 | ♂ 33.7 ±0.2 |  |  |
